# Anticoagulation for Mechanical Aortic Valve in a Patient With Aortic Dissection and Pulmonary Hemorrhage: A Case Report

**DOI:** 10.7759/cureus.69483

**Published:** 2024-09-15

**Authors:** Chenfan Xia, Jiawei Xu, Anmol Bassi

**Affiliations:** 1 Department of Medicine, Frankston Hospital, Melbourne, AUS

**Keywords:** anticoagulation for mechanical valve, aortic dissection (ad), complications of anticoagulation, major bleeding events, mechanical aortic valve, mechanical heart valves, pulmonary haemorrhage, pulmonary hemorrhage, type a aortic dissection

## Abstract

Acute aortic dissections are considered surgical emergencies because they are catastrophic bleeding events. The risk of bleeding is further increased if the patient requires anticoagulation for other comorbidities, such as a mechanical heart valve.

This case study describes a 73-year-old gentleman who presented with massive hemoptysis due to an acute aortic dissection complicated by pulmonary hemorrhage in the context of previous aortic dissection with multiple repair surgeries and residual chronic aortic dissection. He was also on warfarin for a mechanical aortic valve complicated by supratherapeutic international normalized ratio. His acute aortic dissection was treated conservatively without surgery, and he survived. Concerning the risk of thromboembolism from the mechanical aortic valve, anticoagulation was reintroduced one week after his initial bleeding. We changed warfarin to enoxaparin, which was started at a small dose, 40 mg subcutaneously once a day, then gradually increased to the full therapeutic dose, 90 mg (1 mg/kg) twice daily over a week. He was not fully anticoagulated for two weeks. Fortunately, he did not develop any thrombosis. Hemoglobin and Factor Xa levels were closely monitored. He tolerated the enoxaparin without further bleeding.

This type of case is rare and has not been previously reported, considering the patient survived acute aortic dissection with conservative management, did not develop any thrombosis from the mechanical aortic valve when anticoagulation was withheld, and did not experience rebleeding when anticoagulation was restarted. Further research and guidelines are needed to assist clinicians in managing anticoagulation when facing the dilemmas of the risk of bleeding and the risk of thromboembolism. This is particularly important in complex scenarios, such as for patients with mechanical heart valves who subsequently develop contraindications such as aortic dissection or other life-threatening bleeding events.

## Introduction

Aortic dissection is a condition in which the layers of the aortic wall separate [1]. Acute dissections are considered surgical emergencies because they are catastrophic bleeding events [1]. The risk of bleeding is further increased if the patient is on anticoagulation for other comorbidities, such as a mechanical heart valve. One study reported that long-term anticoagulation can be administered safely to patients with repaired type A acute aortic dissection [2]. However, there is missing literature about anticoagulation in unrepaired acute aortic dissection. Additionally, mechanical heart valves require anticoagulation due to the risk of thromboembolism. While there are studies about mechanical heart valves and anticoagulation in intracranial or gastrointestinal bleeding, no data is available specifically for aortic dissection [3]. The management of anticoagulation for mechanical heart valves during life-threatening bleeding is a challenge as the clinician has to balance the risk of bleeding with the risk of thromboembolism. 

This report describes the case of a 73-year-old gentleman with chronic aortic dissection who experienced an acute aortic rupture complicated by pulmonary hemorrhage while on warfarin for a mechanical aortic valve complicated by supratherapeutic international normalized ratio (INR). He was treated conservatively without surgery and survived. He was not fully anticoagulated for two weeks. Fortunately, he did not develop any thrombosis. We changed warfarin to enoxaparin, which was started gradually. He tolerated it well without further bleeding. 

This type of case is rare and has not been previously reported, considering the patient survived acute aortic dissection with conservative management, did not develop any thrombosis from the mechanical aortic valve when anticoagulation was withheld, and did not experience rebleeding when anticoagulation was restarted. It offers valuable insights into managing anticoagulation for mechanical aortic valves in the presence of aortic dissection and highlights the need for further research. 

## Case presentation

A 73-year-old Caucasian male retired gardener presented to the emergency department with an acute onset of massive hemoptysis complicated by hemorrhagic shock. 

Previous aortic dissections and surgeries

He had a Stanford type A aortic dissection 21 years ago, for which he received a mechanical aortic valve and a stent placement to treat the ascending aortic aneurysm. Subsequently, he underwent replacement of the ascending aorta and the arch 18 years ago. However, three years ago, he was found to have chronic aortic dissection after an aortogram. Last year, a computed tomography (CT) angiogram showed complex chronic dissection of the thoracic and abdominal aorta with an aneurysm and penetrating ulcer at the aortic arch, which was increasing in size. Due to the complexity and associated risks, further surgical management was not an option. He had a family history of aortic dissection and mitral valve prolapse but no formal genetic diagnosis of connective tissue disease.

Medication history

He was taking warfarin for the mechanical aortic valve. His INR readings fluctuated, requiring frequent warfarin dose adjustment. One month before the presentation, INR was 1.3 subtherapeutic, and the warfarin dose was increased from 10 mg to 12 mg daily. Two weeks prior to the presentation, his INR was 4.3 supratherapeutic. Warfarin dose was then reduced to 10.5 mg daily. For unclear reasons, he missed his scheduled INR check one week before the presentation. He was also on medications that theoretically may interact with warfarin, including amitriptyline, pantoprazole, and carbamazepine. His other medications include Metoprolol 75 mg twice daily, Irbesartan 300 mg daily, ezetimibe, fluvastatin, dutasteride, and tamsulosin.

Other medical conditions

His other medical problems included long-standing hypertension, depression, gastroesophageal reflux disease, a non-convinced epilepsy history, hyperlipidemia, asymptomatic right frontal stroke, and benign prostate hypertrophy. He quit smoking at the age of 35 and drank alcohol occasionally. Premorbidly, he lived at home independently with his family, had normal exercise tolerance, and still drove.

On presentation, he was hypotensive with a systolic blood pressure of 88 mmHg and tachycardic with a heart rate of 112/minute. He was treated with intravenous fluid. His blood pressure improved to 133/81 mmHg, and his heart rate improved to 89/minute. In addition, he was hypoxic with an oxygen saturation of 80% on room air and tachypnea with a respiratory rate of 28/minute. He required 60% high-flow oxygen at 45 L/minute to maintain an oxygen saturation of 88%. His hemoglobin level was 120 g/L, dropped from a baseline of 150 g/L (Table 1).

**Table 1:** Vital signs and hemoglobin levels during admission

	On presentation	One week after presentation	Two weeks after presentation	Three weeks after presentation
Blood pressure (mmHg)	88/62	106/87	117/73	125/72
Heart rate (per minute)	112	105	91	71
Respiratory rate (per minute)	28	18	18	18
Oxygen saturation (%)	80% on room air	91% on high flow oxygen (40% oxygen with 48 L/minute flow rate)	97% on 2 L/minute nasal prongs	93% on room air
Hemoglobin level (g/L)	120	103	98	108

**Figure 1:**
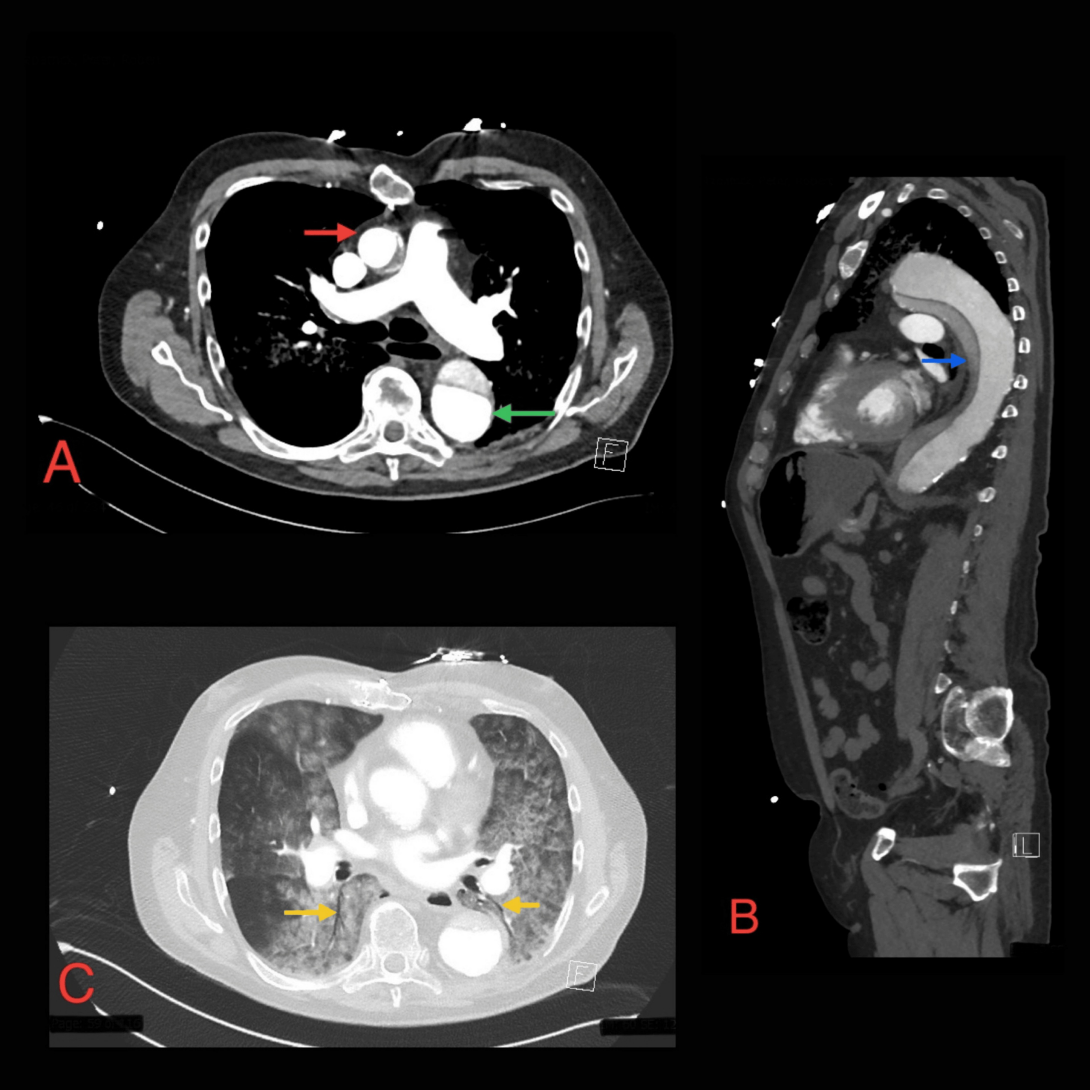
CT angiogram shows aortic dissection with bilateral pulmonary hemorrhage (A) An axial CT image shows aortic dissection affecting both the ascending (red arrow) and descending aorta (green arrow), classifying it as Stanford type A aortic dissection due to the involvement of the ascending aorta. Aortic dissection is associated with a high mortality rate. (B) Aortic dissection (blue arrow) on the sagittal CT image. (C) An axial CT image shows new extensive consolidation and ground glass opacity throughout both lungs with associated air bronchograms (yellow arrows), consistent with extensive pulmonary hemorrhage from acute aortic dissection. Pulmonary hemorrhage is a fatal complication of Stanford type A aortic dissection.

The INR was supratherapeutic at 3.9, which was reversed by prothrombinex. CT angiogram, in comparison with the previous image, showed that the chronic aortic ulcer appeared larger and inflamed, with aortic rupture into the adjacent left upper lobe of the lung and extensive new bilateral pulmonary hemorrhage, as well as blood in the stomach, suggesting an acute rupture, which is consistent with the sudden onset of hemoptysis (Figure 1).

Due to the complexity of his aortic disease, there is a high risk of spinal cord ischemia if surgical repair is attempted. Therefore, elective or emergency repair surgeries were not considered viable options. Palliative supportive care was decided to be the most appropriate approach. He was alert and oriented at the time. High-flow oxygen and intravenous fluids were continued. Regular medications were ceased. He also received subcutaneous morphine and midazolam as required for comfort, which was changed to a syringe driver later on.

In the following days, he experienced intermittent hemoptysis but no major bleeding. His hemoglobin level remained stable. His vital signs improved, and he required less oxygen. He also had an episode of fever, most likely from pneumonia, which was successfully treated with a course of intravenous antibiotics, ceftriaxone and azithromycin.

Due to the concern of thrombosis risk and balancing it with the risk of further bleeding from aortic dissection or pulmonary hemorrhage, enoxaparin was started on day seven of admission at a small dose of 40 mg subcutaneously once a day. We changed warfarin to enoxaparin as he was experiencing difficulty with maintaining a therapeutic range of INR. The supratherapeutic INR on presentation was contributing to his life-threatening bleeding. Enoxaparin dose was increased to 90 mg daily (1 mg/kg) on day 14 and further increased to a full therapeutic anticoagulation dosage of 90 mg twice daily on day 15. Factor Xa and hemoglobin level were closely monitored to watch for rebleeding and ensure the therapeutic range of enoxaparin. Fortunately, he tolerated enoxaparin well without further bleeding. 

He was hospitalized for a total of three weeks and then discharged home. He planned to follow up with the community care team to monitor hemoptysis, Factor Xa, and hemoglobin levels and have a repeat CT chest with an outpatient clinic review in one month. 

## Discussion

Aortic dissection is associated with a high mortality rate, with at least 30% of patients dying upon reaching the emergency room [1]. Post-surgery, mortality rates range between 20% and 30% [1]. In addition, spinal cord ischemia is a complication after the repair of an aortic aneurysm or dissection [4]. The rate is around one in 130 for patients undergoing aortic dissection or ruptured aortic aneurysm repair and one in 600 for patients undergoing unruptured aortic aneurysm repair [4]. Hemorrhage extending to the pulmonary artery is a fatal complication of Stanford type A aortic dissection [5]. One study reported three cases of aortic dissection with pulmonary hemorrhage [5]. Two patients died while waiting for emergency surgery despite resuscitation efforts [5]. The third patient was not considered for surgery due to advanced age and comorbidities and was transferred to a nursing home for conservative management, for which the report did not mention the outcome [5]. Possibly, the patient left the hospital, and no follow-up information was available in the hospital system at the time of the study.

Anticoagulation is indicated for patients with mechanical heart valves due to the risk of valve thrombosis and systemic thromboembolism [6]. One study showed that the risk of major embolism without anticoagulation is four per 100 patient-years [6]. Interestingly, there are a few case reports of individuals with mechanical heart valves who were not anticoagulated. The longest event-free survival for a mechanical aortic valve was 37 years, and for a mechanical mitral valve, it was 27 years [7,8]. One research suggested that the management of life-threatening bleeding in patients on warfarin for mechanical heart valves should include vitamin K, fresh frozen plasma, or prothrombin complex concentrate [3].

Our patient experienced an acute aortic dissection with pulmonary hemorrhage in the context of a pre-existing chronic aortic dissection. Additionally, the patient was on warfarin with a supratherapeutic INR, which further increased the bleeding risk. He received prothrombinex for warfarin reversal. His bleeding stopped spontaneously, with no recurrence even after initiating enoxaparin, which is unusual, and the underlying mechanism is unclear. Furthermore, he was not fully anticoagulated for two weeks and did not develop any thrombosis.

It is well known that a mechanical aortic valve without anticoagulation is associated with a high risk of thromboembolism. While there are no guidelines available at present regarding the safe duration to withhold anticoagulants in the event of major bleeding, one study suggests that anticoagulation could be safely stopped for one to two weeks with minimal risk of thromboembolism [3,9]. In our particular case, the patient did not receive full anticoagulation for two weeks.

Fatal rebleeding after resuming anticoagulation is a risk that must be balanced with thrombosis risk. There are currently no guidelines regarding the safe approach of resumption anticoagulation, and data is limited to only case reports [3]. In our case, we decided to reintroduce anticoagulation gradually. Enoxaparin was used with closely monitored Factor Xa and hemoglobin levels to ensure the therapeutic range. Determining when and how to restart anticoagulation was challenging for clinical decision-making. Additionally, it might be debatable whether we should continue anticoagulation given the ongoing major bleeding risk.

## Conclusions

This case study describes a 73-year-old gentleman who suffered an acute aortic dissection complicated by pulmonary hemorrhage in the context of a pre-existing chronic aortic dissection and the use of warfarin for a mechanical aortic valve. Fortunately, the bleeding stopped spontaneously without surgery. Anticoagulation with enoxaparin was gradually reintroduced, and the patient tolerated it well without rebleeding. Although he was not fully anticoagulated for two weeks, he did not develop any thromboembolic event. 

While this is a rare case, it provides valuable insights into managing anticoagulation during life-threatening bleeding events. Guidelines are needed to assist clinicians in managing anticoagulation for patients with mechanical heart valves who subsequently develop contraindications such as aortic dissection or other major bleeding problems. 

We restarted anticoagulation with the choice of enoxaparin one week after the initial bleeding at a small dose of 40 mg daily, then gradually increased to a full therapeutic dose of 1 mg/kg twice a day over a week. We closely monitored hemoglobin levels to watch for rebleeding and Factor Xa levels to ensure the therapeutic range of enoxaparin. This strategy worked well for our patient. However, more evidence is required to assess whether it will be effective for other similar clinical scenarios. Further research, such as retrospective cohort studies, would be helpful to address this gap. 
